# 3D Adversarial Segmentation of Kidney-Transplant Across Multiple MRI Sequences Using Probabilistic and Anatomical Priors

**DOI:** 10.3390/diagnostics16091369

**Published:** 2026-04-30

**Authors:** Israa Sharaby, Ahmed Alksas, Hossam Magdy Balaha, Ali Mahmoud, Mohammed Badawy, Mohamed Abou El-Ghar, Mohammed Ghazal, Asem M. Ali, Moumen El-Melegy, Sohail Contractor, Ayman El-Baz

**Affiliations:** 1 Bioengineering Department, University of Louisville, Louisville, KY 40292, USA; 2 Radiology Department, Urology and Nephrology Center, Mansoura University, Mansoura 35516, Egypt; 3 Electrical, Computer, and Biomedical Engineering Department, Abu Dhabi University, Abu Dhabi 59911, United Arab Emirates; 4 Electrical Engineering Department, Assiut University, Assiut 71516, Egypt; 5 Department of Radiology, University of Louisville, Louisville, KY 40202, USA

**Keywords:** kidney-transplant, MRI segmentation, BOLD-MRI, DW-MRI, probabilistic appearance prior, anatomical prior, generative adversarial network

## Abstract

**Background/Objectives**: Accurate kidney segmentation from magnetic resonance imaging (MRI) in kidney-transplant patients is essential for quantitative graft assessment, yet it remains challenging due to low tissue contrast, intensity inhomogeneity, and inter-patient anatomical variability introduced by surgical graft placement. **Methods**: We propose a 3D adversarial segmentation framework that incorporates probabilistic appearance and anatomical shape priors into a residual conditional generative adversarial network (GAN). The framework integrates image-driven and prior-guided information to improve boundary delineation under challenging imaging conditions and is evaluated on 100 kidney-transplant patients across T2-weighted imaging, BOLD-MRI, and DW-MRI using leave-one-out cross-validation. **Results**: The proposed method achieves mean Dice scores of 90.86% on T2-weighted imaging, 92.02% on BOLD-MRI, and 94.00% on DW-MRI. Consistent performance across all modalities demonstrates robustness under heterogeneous MRI acquisitions. The incorporation of prior guidance improves segmentation stability and anatomical consistency, particularly in low-contrast modalities. **Conclusions**: The proposed framework enables reliable kidney delineation across multiple MRI sequences, supporting consistent extraction of quantitative imaging biomarkers. This capability facilitates noninvasive assessment of renal graft function and supports longitudinal monitoring of transplant patients.

## 1. Introduction

**Figure 1:**
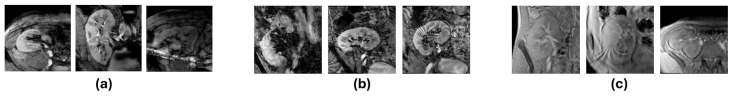
Key challenges in renal MRI segmentation: (**a**) intricate kidney shape in kidney transplant patients, (**b**) noise in MRI scans, and (**c**) low contrast and similar intensity distributions between renal tissues and surrounding structures.

Kidney transplantation is the definitive treatment for end-stage kidney disease, offering better outcomes and quality of life than prolonged dialysis [1]. However, the post-transplant period remains vulnerable to both acute rejection in the early phase and chronic allograft injury driven by fibrotic processes in the long term, making early and reliable assessment essential for preserving graft function [2,3]. Automated kidney segmentation is an essential prerequisite for quantitative kidney measurements because inaccurate delineation may propagate errors into downstream biomarker estimation and confound clinical interpretation [4]. Automated kidney segmentation in transplant magnetic resonance imaging (MRI), however, remains an open problem that presents different challenges from those encountered in native kidney segmentation, as shown in Figure 1. Surgical graft placement introduces substantial variability in kidney orientation, position, and morphology across patients, while MRI exhibits heterogeneous intensity distributions, noise, and low contrast between renal parenchyma and surrounding tissues, resulting in ambiguous boundaries that are difficult to delineate reliably [5,6].

GAN-based segmentation studies in renal imaging have focused mainly on CT because CT provides relatively high and standardized tissue contrast via Hounsfield Units and more stable intensity distributions across scanners and institutions [7]. Shan et al. [8] enhanced kidney segmentation in CT by incorporating densely interlinked structures into the generator of SegTGAN, yielding a Dice coefficient of 0.92 and surpassing conventional U-Net baselines. Ruan et al. [9] proposed a multi-branch feature-sharing GAN for kidney tumor segmentation in CT, achieving a Dice coefficient of 0.85 and demonstrating advantages over U-Net variants. Eslami et al. [10] addressed low-dose CT denoising and segmentation jointly using a conditional GAN, achieving a Dice score of 0.79, which outperformed both U-Net and WGAN. Although these studies demonstrate the effectiveness of adversarial learning, they were validated under imaging conditions that are more favorable than those encountered in kidney-transplant MRI.

Another line of work has attempted to bridge modality gaps through cross-modality transfer learning. Guo et al. [11] used CycleGAN to generate synthetic MRI from T2-weighted acquisitions, achieving Dice values between 0.91 and 0.93; Song et al. [12] applied CycleGAN for CT-to-ultrasound domain transfer, reporting Dice scores between 0.826 and 0.853; Causey et al. [13] trained Mask-RCNN and U-Net on the KiTS19 benchmark, achieving Dice scores of 0.94 and 0.60, respectively, and Pang et al. [14] proposed CTumorGAN for CT-based tumor segmentation, achieving a Dice score of 0.83. Despite their promise, these methods remain anchored to high-contrast domains and do not address the low-contrast, multisequence, and anatomically variable setting of kidney-transplant MRI. Despite the predominance of CT-based approaches, MRI is uniquely suited to transplant assessment. MRI does not involve ionizing radiation and avoids the nephrotoxic risks associated with iodinated contrast agents [15], making it better suited for repetitive and long-term monitoring of impaired renal function. Critically, renal MRI protocols acquire multiple complementary sequences that encode distinct physiological properties of the transplanted kidney. These sequences collectively provide noninvasive insight into renal graft pathophysiology, enabling the assessment of structural integrity, tissue oxygenation, and microstructural alterations [2,3,16,17,18,19]. T2-weighted imaging (T2WI) provides morphological information with high spatial resolution but inherently low parenchyma-to-background contrast. Blood-oxygen-level-dependent MRI (BOLD-MRI), acquired with multiple echo times, reflects alterations in tissue oxygenation and perfusion but introduces signal decay and susceptibility artifacts that complicate segmentation. Diffusion-weighted MRI (DW-MRI), acquired across multiple b-values, captures microstructural changes related to cellular integrity, filtration properties, and inflammation [20]. In addition, T1-weighted imaging and T1 mapping have been shown to be associated with fibrosis and chronic structural remodeling in renal allografts, providing complementary information on long-term graft injury [2,3,19]. Together, these MRI modalities enable a comprehensive, noninvasive evaluation of key physiological processes in the transplanted kidney, including blood flow, filtration, inflammation, and fibrosis. However, T1-based measurements primarily reflect structural remodeling, whereas functional MRI sequences such as BOLD-MRI and DW-MRI are more sensitive to early physiological changes in the transplanted kidney [16,17]. In routine renal MRI protocols, T2-weighted imaging is widely used as the anatomical reference for assessing kidney morphology and volume [18]. Therefore, this study focuses on T2WI along with BOLD-MRI and DW-MRI to enable segmentation across modalities directly used for integrated structural and functional assessment of the renal allograft, ensuring consistency for downstream quantitative biomarker analysis.

**Figure 2:**
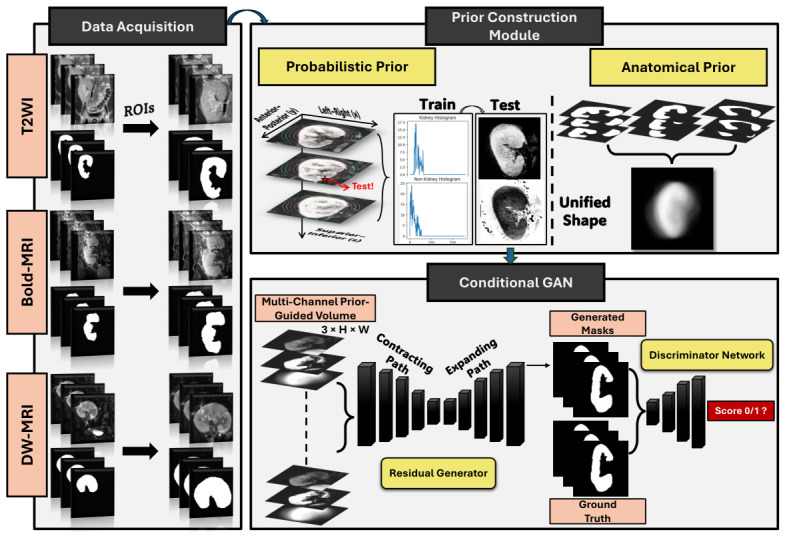
An overview of the proposed prior-guided renal segmentation framework. Each MRI modality is processed independently. First, probabilistic appearance prior and an anatomical shape prior are constructed then concatenated with the ROI image to form a three-channel condition volume that is provided to the conditional GAN to generate the mask volume.

Independent analysis of the quantitative biomarkers derived from each of these sequences, including morphological characteristics, tissue oxygenation, and diffusion-related microstructural properties, is essential for comprehensive graft assessment. Reliable segmentation must, therefore, generalize across all three modalities to enable consistent biomarker extraction. Goyal et al. [21] used Mask R-CNN for kidney segmentation in T2-weighted MRI, reporting a Dice score of 0.90, but focused on a single modality and did not investigate performance across different MRI sequences. Liang et al. [22] applied nn-UNet on DWI and BOLD sequences, achieving Dice scores ranging from 0.86 to 0.92, but did not address the anatomical variability and low-contrast boundary challenges specific to the transplant setting. These studies highlight the clinical value of multiparametric renal MRI while also underscoring the need for segmentation frameworks that remain robust across heterogeneous MRI sequences. Generative adversarial networks (GANs) offer a promising solution to these challenges, as they replace traditional pixelwise loss functions with an adversarial objective that encourages anatomically plausible outputs and penalizes structurally inconsistent predictions [9,23,24]. However, extensive examination of GAN-based segmentation under the specific challenges of transplant renal MRI remains limited. To address these limitations, we propose a 3D adversarial segmentation framework illustrated in Figure 2 that integrates two prior representations directly into the conditional input of a residual conditional GAN and generalizes across T2WI, BOLD-MRI, and DW-MRI. The main contributions of this work are as follows:

1. We propose a prior-guided 3D adversarial segmentation framework for kidney-transplant MRI that embeds probabilistic appearance map and anatomical shape prior as conditioning inputs. The framework is evaluated on 100 kidney-transplant patients each with T2WI, BOLD-MRI, and DW-MRI using leave-one-out cross-validation.
2. We derive a voxelwise probabilistic appearance model based on contour-level intensity histograms encoding foreground and background likelihoods as a function of both intensity and spatial position, incorporating adjacent-slice context to capture local 3D structure.
3. We construct a spatially varying anatomical shape prior derived from the cohort masks, providing a structural guidance channel that is independent of image intensity and constrains the network toward anatomically consistent predictions.
4. We conduct a comprehensive evaluation across T2WI, BOLD-MRI, and DW-MRI to investigate the influence of each modality-specific imaging characteristics influence segmentation performance and identify which modality yields the most reliable kidney segmentation.

## 2. Materials: Dataset

This study was performed with institutional review board approval (IRB#14.1052) and all of the subjects provided written informed consent before imaging. The dataset includes multisequence renal MRI images from 100 kidney-transplant patients suspected of renal allograft rejection at the Urology and Nephrology Center, Mansoura University, Mansoura, Egypt. All tests were performed with a 3 T Philips Ingenia scanner (Philips Medical Systems, Best, The Netherlands). For each subject, three MRI sequences were obtained: T2-weighted imaging (T2WI), blood-oxygen-level-dependent MRI (BOLD-MRI), and diffusion-weighted MRI (DW-MRI). The T2WI sequence, which has high spatial resolution and innate low parenchyma-to-background contrast, is a kind of morphology. The BOLD-MRI sequence uses a multi-echo technique by five echo times in order to investigate renal oxygenation. It introduces signal decay and susceptibility artifacts that make segmentation challenging. The DW-MRI sequence was acquired in eleven *b*-values from 0 to 1000 s/mm^2^. For segmentation experiments, the b = 400 s/mm^2^ volume was taken as a representative diffusion-weighted acquisition. This intermediate b-value allowed stable contrast of the kidney and background while maintaining an adequate signal-to-noise ratio to allow reliable delineation of the mask. Acquisition parameters for the three sequences are summarized in Table 1.

Ground-truth kidney masks were delineated manually for each subject by radiologists with dedicated experience in renal imaging, and were used as the reference standard for both network training and performance evaluation.

## 3. Methods

The proposed framework is implemented independently for each of the MRI modalities (T2WI, BOLD-MRI, DW-MRI), utilizing the same processing pipeline. Two prior representations for a given modality are created from the available data of that modality. First, we have a voxelwise probabilistic appearance model estimating the likelihoods of foreground and background intensities through contour-level histogram statistics. The second one is an anatomical shape prior that is computed by voxelwise averaging of the corresponding binary masks to capture the spatial distribution of the kidney within that modality. Both priors are then concatenated with the raw grayscale MRI volume of the same modality, yielding a three-channel condition volume. This condition volume provides the basis for a 3D residual conditional generative adversarial network (GAN) that performs volumetric kidney segmentation.

### 3.1. Problem Formulation

Let $\Omega \subset Z^{3}$ denote the discrete 3D lattice of voxel coordinates supporting a volumetric MRI scan, where each voxel location is represented by $x=(x_{1},x_{2},x_{3})\in \Omega$. The spatial dimensions of the lattice are denoted by D×H×W, corresponding to the depth, height, and width of the volume.

For a given MRI modality, let g:Ω→R denote the observed grayscale volume, where g(x) is the intensity at voxel x. Let m:Ω→{0,1} denote the corresponding binary segmentation map, where m(x)=1 indicates kidney foreground and m(x)=0 indicates background. The objective of segmentation is to estimate *m* from the observed image *g*.

To enhance boundary localization under low-contrast and heterogeneous acquisition conditions, we augment the raw image with two complementary spatial priors defined over the same lattice Ω. The first prior is a spatially variant voxelwise probabilistic appearance map $P_{\mathrm{app}}:\Omega \to \left[0,1\right]$, where $P_{\mathrm{app}}\left(x\right)$ denotes the estimated likelihood that voxel x belongs to the kidney foreground, conditioned on its observed intensity and its contour-band assignment. This map encodes localized intensity statistics derived from the training data, and therefore, varies spatially across the volume. The second prior is a data-driven anatomical shape map S:Ω→[0,1], defined such that S(x) equals the voxelwise average of the ground-truth masks at location x, capturing the spatial distribution of the kidney within the cohort.

The three volumetric components are the image volume *g*, the appearance prior $P_{\mathrm{app}}$, and the anatomical map S concatenated to form a three-channel condition volume $c=\left(g,P_{\mathrm{app}},S\right)\in R^{3\times D\times H\times W}$, which serves as the input to the 3D adversarial segmentation network. The network produces the final segmentation estimate $\hat{m}:\Omega \to \left\{0,1\right\}$. The construction of each prior and the adversarial learning objective are described in the following subsections.

### 3.2. Voxelwise Probabilistic Appearance Prior

**Figure 3:**
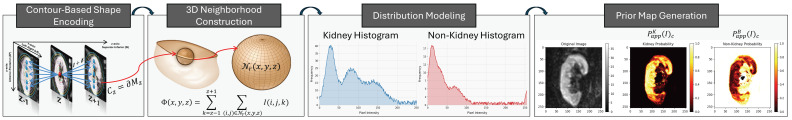
Pipeline for voxelwise appearance prior construction. For each slice *z*, the kidney boundary contour $C_{z}=\partial M_{z}$ is extracted and a 3D neighborhood $N_{r}\left(x,y,z\right)$ is constructed by aggregating intensity from adjacent slices. Kidney and background intensity histograms are computed from the aggregated neighborhoods and used to generate the voxelwise appearance map $P_{\mathrm{app}}$, which forms channel $c_{2}$ of the condition volume.

This formulation builds upon the contour-based intensity modeling framework introduced in [25], where iso-contour bands are used to capture spatially varying intensity characteristics. In the original 2D formulation, histogram statistics were aggregated independently within each slice. Here, the framework represented in Algorithm 1 is extended to 3D by incorporating adjacent-slice information, allowing intensity evidence to be accumulated within a volumetric neighborhood consistent with the three-dimensional nature of the segmentation network. The resulting appearance map constitutes the second channel $c_{2}$ of the condition volume. The overall procedure is illustrated in Figure 3.
**Algorithm 1** Voxelwise Appearance Prior Map Construction
**Training:** Given ${\left\{g^{\left(n\right)},M^{\left(n\right)}\right\}}_{n=1}^{N}$, initialize $H_{c}^{K}\left(I\right),H_{c}^{B}\left(I\right)\leftarrow 0$ for all $c\in \{1,\ldots ,N_{c}\}$1:**for** each subject *n*, slice *z*, contour band *c* **do**2:   Extract contour $C_{z}=\partial M_{z}$ and partition $\Omega_{z}$ into $N_{c}$ iso-contour bands3:   Construct $N_{r}\left(x,y,z\right)$ and compute Φ(x,y,z)4:   Update $H_{c}^{K}\left(I\right)$ and $H_{c}^{B}\left(I\right)$ using kidney/background voxels in band *c*5:**end for**
**Output:** ${\left\{H_{c}^{K}\left(I\right),H_{c}^{B}\left(I\right)\right\}}_{c=1}^{N_{c}}$
**Inference:** Given g and ${\left\{H_{c}^{K}\left(I\right),H_{c}^{B}\left(I\right)\right\}}_{c=1}^{N_{c}}$6:**for** each voxel with intensity *I* in contour band *c* **do**7:   **if** $H_{c}^{K}\left(I\right)+H_{c}^{B}\left(I\right)>0$ **then**8:   $P_{\mathrm{app}}^{K}{\left(I\right)}_{c}=\frac{H_{c}^{K}\left(I\right)}{H_{c}^{K}\left(I\right)+H_{c}^{B}\left(I\right)}$9:   **else**10:   Expand I±δ, δ∈{1,2,3}; if no support found, set $P_{\mathrm{app}}^{K}{\left(I\right)}_{c}=0.5$11:   **end if**12:**end for**
**Output:** $P_{\mathrm{app}}^{K}{\left(I\right)}_{c}\to$ channel $c_{2}$

Let $\Omega_{z}\subset Z^{2}$ represent the discrete 2D lattice of pixel coordinates at slice index *z*, and $M_{z}:\Omega_{z}\to \left\{0,1\right\}$ represent the corresponding binary mask. We define the kidney boundary contour at slice *z* as $C_{z}=\partial M_{z}$. Each slice is partitioned into $N_{c}$ concentric iso-contour bands of fixed thickness originating from the kidney centroid. The $c\in \{1,\ldots ,N_{c}\}$ bands define a spatially localized region in which the kidney and background intensity statistics are modeled independently. This contour-based partitioning accounts for spatial variation in the intensity distributions, which cannot be expressed by a global model.

Using each voxel (x,y,z) belonging to contour band *c*, a 3D spatial neighborhood $N_{r}\left(x,y,z\right)$ is established containing spatially adjacent voxels within the same contour band for slices z−1, *z*, and z+1. A total intensity response obtained from the neighborhood is presented as follows:

(1) $$\Phi \left(x,y,z\right)=\sum_{k=z-1}^{z+1}\sum_{\left(i,j\right)\in N_{r}\left(x,y,z\right)}I\left(i,j,k\right),$$

where I(i,j,k) denotes the intensity at voxel (i,j,k). The kidney and background intensity histograms from Φ are accumulated for each contour band *c*, across all subjects. We denote histogram counts corresponding to kidney and background voxels at intensity level *I* as $H_{c}^{K}\left(I\right)$ and $H_{c}^{B}\left(I\right)$ for each contour band *c*. These histograms are then aggregated across subjects to obtain contour-specific intensity models ${\left\{H_{c}^{K}\left(I\right),H_{c}^{B}\left(I\right)\right\}}_{c=1}^{N_{c}}$.

Based on the learned histograms, the appearance score for an observed intensity value *I* in contour band *c* is computed as the kidney likelihood

(2) $$P_{\mathrm{app}}^{K}{\left(I\right)}_{c}=\frac{H_{c}^{K}\left(I\right)}{H_{c}^{K}\left(I\right)+H_{c}^{B}\left(I\right)}.$$

Here, $H_{c}^{K}\left(I\right)$ and $H_{c}^{B}\left(I\right)$ denote the histogram counts of intensity *I* for the kidney and background classes, respectively, within contour band *c*. The resulting $P_{\mathrm{app}}^{K}{\left(I\right)}_{c}$ measures the kidney intensity distribution compared with the background distribution in the corresponding spatial band. Values close to one indicate strong agreement with the kidney appearance statistics, while values close to zero indicate stronger support for the background class.

For $H_{c}^{K}\left(I\right)+H_{c}^{B}\left(I\right)=0$, the intensity lookup is expanded to neighboring gray-level values I±δ, with δ incremented progressively up to a maximum tolerance of 3 until non-zero histogram support is found. If no support is observed within this tolerance range, the voxel is assigned $P_{\mathrm{app}}^{K}{\left(I\right)}_{c}=0.5$, reflecting the absence of discriminative intensity evidence for that contour band. The resulting voxelwise map $P_{\mathrm{app}}^{K}{\left(I\right)}_{c}$ forms channel $c_{2}$ of the condition volume and provides spatially localized appearance guidance that complements the raw grayscale input $c_{1}$.

### 3.3. Data-Driven Anatomical Shape Prior

Shape priors are introduced into segmentation frameworks through multiple paradigms including atlas-based registration models, statistical shape models, and learnable prototype-based approaches [26]. Nonetheless, these paradigms share a singular formulation, wherein explicit structural knowledge is combined with the image evidence to constrain the segmentation prediction. Based on You et al. [26], the process can be written as the following:

(3) $$\hat{m}=F\left(g,\;S(\left\{g^{\left(n\right)}\right\},\;\left\{M^{\left(n\right)}\right\})\right),$$

where F is the segmentation model, g:Ω→R is the input MRI volume, and S represents the anatomical shape prior estimated from reference sets ${\left\{g^{\left(n\right)}\right\}}_{n=1}^{N}$ and their corresponding ground-truth masks ${\left\{M^{\left(n\right)}\right\}}_{n=1}^{N}$. Thus, the prior S encodes structural information derived from the reference cohort, regardless of the input volume g.

In the transplanted kidney MRI, structural constraints, such as those mentioned above, are specifically useful when surgical placement of the graft requires wide variations in both its position and orientation, and the low tissue contrast results in insufficient boundary evidence. Parametric shape models, which represent anatomy as set landmarks or fixed basis functions, are inappropriate in this context since the geometric concept assumed in such shapes is not flexible enough against large variability in transplant cohort morphology. So we instantiate S using Algorithm 2 as a spatially variant anatomical map, directly derived from the ground-truth masks of the training subjects, and not with any parametric form imposed on the underlying anatomy. Therefore, let ${\left\{M^{\left(n\right)}\right\}}_{n=1}^{N}$ be the set of *N* ground-truth binary masks in the current cross-validation fold, where every $M^{\left(n\right)}:\Omega \to \left\{0,1\right\}$ corresponds to a kidney or background label for each voxel. The prior anatomical shape S(x) is constructed by computing the voxelwise average of all training masks:

(4) $$S\left(x\right)=\frac{1}{N}\sum_{n=1}^{N}M^{\left(n\right)}\left(x\right),\;x\in \Omega .$$

**Figure 4:**
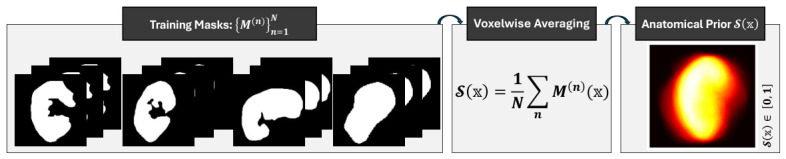
Construction of the anatomical shape prior S from the DW-MRI training masks. Sample binary masks ${\left\{M^{\left(n\right)}\right\}}_{n=1}^{N}$ illustrate the inter-subject variability in kidney shape and position across the cohort. Voxelwise averaging of all masks yields S(x)∈[0,1], where bright regions correspond to voxels labeled as kidney across subjects and darker boundaries reflect anatomical variability.

At any voxel x, S(x)∈[0,1] corresponds to the fraction of training subjects for which that location is labeled as kidney foreground. Voxels consistently labeled as kidney across the training subjects receive values approaching unity, background voxels receive values approaching zero, and anatomically variable boundary regions receive intermediate values representing the structural variability across subjects. The resulting map encodes the common spatial characteristics of the kidney as observed across the cohort, highlighting the anatomical region within which the graft is most consistently located and providing the network with a spatially graded structural reference that is entirely independent of image intensity. Because S is computed independently for each MRI modality and re-estimated at each cross-validation fold, it captures the modality-specific spatial extent of the kidney under each acquisition protocol while remaining adaptive to the training cohort at each fold. The anatomical shape prior S shown in Figure 4 forms the third channel $c_{3}$ of the condition volume, providing the adversarial network with explicit structural guidance that directly complements the appearance-based channel $c_{2}$ derived in Section 3.2.
**Algorithm 2** Anatomical Shape Prior Construction
**Input:** Training masks ${\left\{M^{\left(n\right)}\right\}}_{n=1}^{N}$ for the current cross-validation fold
**Output:** Anatomical shape prior S forming channel $c_{3}$1:Initialize S(x)←0, ∀ x∈Ω2:**for** each training subject n∈{1,…,N} **do**3:   Accumulate: $S\left(x\right)\leftarrow S\left(x\right)+M^{\left(n\right)}\left(x\right)$, ∀ x∈Ω4:**end for**5:Average: $S\left(x\right)\leftarrow \frac{1}{N}S\left(x\right)$, ∀ x∈Ω6:Compute separately for each modality using its corresponding segmentation volume7:Assign S as channel $c_{3}$ of the condition volume for the test subject

### 3.4. 3D Residual Pix2Pix Network for Prior-Conditioned Segmentation

The probabilistic appearance maps and anatomical shape prior derived in Section 3.2 and Section 3.3 offer complementary structural guidance but are both insufficient on their own to obtain accurate volumetric kidney masks. The appearance maps encode spatially localized intensity likelihoods but are more sensitive to inter-sequence differences; the anatomical prior encodes structural constraints, but has no information concerning specific images to be segmented. For unifying the two sources of guidance into a unified segmentation framework, we use a 3D Pix2Pix conditional GAN [27] consisting of generator *G* and a discriminator *D*, and takes the three-channel condition volume $c=[c_{1},c_{2},c_{3}]$ as input to create a binary volumetric kidney mask $\hat{y}$. The generator is augmented with residual decoding blocks to enhance gradient flow and preserve fine spatial detail across the encoder-decoder bottleneck. The adversarial loss $L_{\mathrm{adv}}$ [28] encourages the network to generate masks that are anatomically plausible, thereby penalizing fragmented or structurally inconsistent predictions, which a pixelwise loss [28,29] alone would not sufficiently deter.

**Generator: Residual 3D U-Net**

The generator *G* is a residual 3D U-Net with a fully volumetric encoder–decoder architecture. The convolutional unit is composed of a 3×3×3 Conv3D layer followed by batch normalization [30] and ReLU activation [31]. To compensate for the anisotropic voxel spacing found in MRI acquisitions, wherein in-plane resolution is typically finer than the slice thickness, all downsampling and upsampling operations are treated anisotropically, yielding a spatial stride factor of (1,2,2), allowing the slice dimension to be maintained while in-plane resolution reduction is achieved progressively. At the bottleneck, the encoder increases the number of feature channels from 64 up to 512. Spatial resolution is re-established for the decoder via trilinear upsampling and residual decoding blocks; skip connections from the encoder levels provide fine-grained spatial information at all scales. The generator converts condition volume to a logit mask volume ${\hat{y}}_{\mathrm{logit}}=G\left(c\right)$, so that the final mask $\hat{y}$ is obtained at inference by the sigmoid activation and thresholding at 0.5.

**Discriminator: Conditional 3D PatchGAN**

The discriminator *D* is a conditional 3D PatchGAN [27] discriminator which accepts the mask and condition volume concatenation along the channel dimension as input, generating a volumetric logit grid instead of a scalar score to steer the generator to produce locally consistent predictions at every spatial location. Each discriminator block has a 3×3×3 Conv3D layer, batch normalization, ReLU activation, and the same anisotropic downsampling factors as the generator (1,2,2). The number of feature channels increases progressively to a maximum of 256 before the final 3×3×3 convolution that generates the single-channel logit grid.

**Adversarial Learning Objective**

The discriminator is trained to differentiate real and generated masks, having $L_{D}$ as follows:

(5) $$L_{D}=\frac{1}{2}\left(L_{\mathrm{adv}}\left(D(y,c),1\right)+L_{\mathrm{adv}}\left(D(\hat{y},c),0\right)\right),$$

where **1** and **0** are grids of ones and zeros matching the output resolution, and $L_{\mathrm{adv}}$ is the binary cross-entropy with logits loss [32]. The generator loss $L_{G}$ combines the adversarial term with an $\ell_{1}$ reconstruction term [33]:

(6) $$L_{G}=L_{\mathrm{adv}}\left(D(\hat{y},c),1\right)+\lambda_{\mathrm{rec}}{∥\hat{y}-y∥}_{1},$$

when $\lambda_{\mathrm{rec}}=200$ under all experiments. The reconstruction loss guarantees voxelwise fidelity to the ground truth and the adversarial loss imposes global structural coherence, which a pixelwise objective by itself cannot capture.

## 4. Results

### 4.1. Experimental Protocol

**Figure 5:**
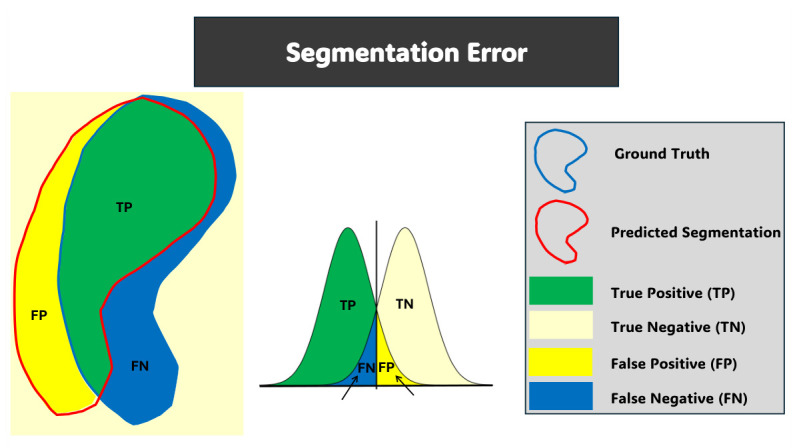
Conceptual illustration of segmentation error analysis. The overlap between ground truth and predicted segmentation defines TP, TN, FP, and FN regions.

All experiments were conducted in PyTorch 2.6.0 and trained for 200 epochs with the Adam optimizer, learning rate $4\times 10^{-4}$, and batch size 1 on an NVIDIA GeForce RTX 4080 GPU. The same network architecture, loss formulation, and training procedure were used for all MRI modalities. Each modality had its own appearance prior, shape prior, and the respective image volumes. Performance analysis was performed with leave-one-out cross-validation (LOOCV) on each modality. A subject was held out for each fold for evaluation, with the remaining subjects in each fold partitioned into 80% for training and 20% for validation. The performance of segmentation was evaluated with four different metrics to end the full framework described in Algorithm 3. These metrics are as follows: Dice similarity coefficient (DSC) and intersection-over-union (IoU), for volumetric overlap computation, and the 95th percentile Hausdorff distance (HD95) and average symmetric surface distance (ASSD) to measure boundary accuracy. We calculate those metrics based on the voxelwise agreement between predicted masks and reference masks as illustrated in Figure 5.
**Algorithm 3** The guided 3D adversarial segmentation framework applied independently per MRI modality
**Input:** For a modality M∈{T2WI,BOLD,DW(b=400)}, volumes ${\left\{g^{\left(n\right)}\right\}}_{n=1}^{N}$ and masks ${\left\{M^{\left(n\right)}\right\}}_{n=1}^{N}$
**Output:** Predictions $\left\{{\hat{M}}^{\left(n\right)}\right\}$ and LOOCV metrics (mean ± std)1:**for** each modality M **do**2:   **for** s=1 to *N* **do**▹ LOOCV fold: hold out subject *s*3:   Define reference set R={1,…,N}∖{s}4:   **ROI standardization:** extract kidney-centered ROI for all subjects5:   **Appearance prior:** build contour-band histograms from ${\left\{\left(g^{\left(n\right)},M^{\left(n\right)}\right)\right\}}_{n\in R}$ and compute $P_{\mathrm{app}}$ for subject *s* (Algorithm 1)6:   **Shape prior:** compute anatomical map S from ${\left\{M^{\left(n\right)}\right\}}_{n\in R}$ (Algorithm 2)7:   **Condition volume:** form $c=[\;g,\;P_{\mathrm{app}},\;S\;]$8:   **Train 3D conditional GAN:** optimize *G* and *D* using Equations (5) and (6)9:   **Inference:** ${\hat{M}}^{\left(s\right)}\leftarrow G\left(c^{\left(s\right)}\right)$ and threshold at 0.510:   **Evaluation:** compute DSC, IoU, HD95, ASSD between ${\hat{M}}^{\left(s\right)}$ and $M^{\left(s\right)}$11:   **end for**12:   Report modality-specific LOOCV results (mean ± std)13:**end for**

### 4.2. Ablation Study

**Figure 6:**
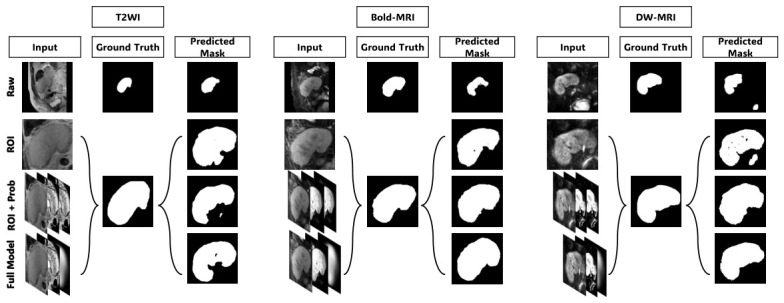
Representative axial slice from the predicted volumetric masks under each ablation configuration across the three MRI modalities. Each group shows the input, ground-truth mask, and predicted mask for a representative subject.

**Figure 7:**
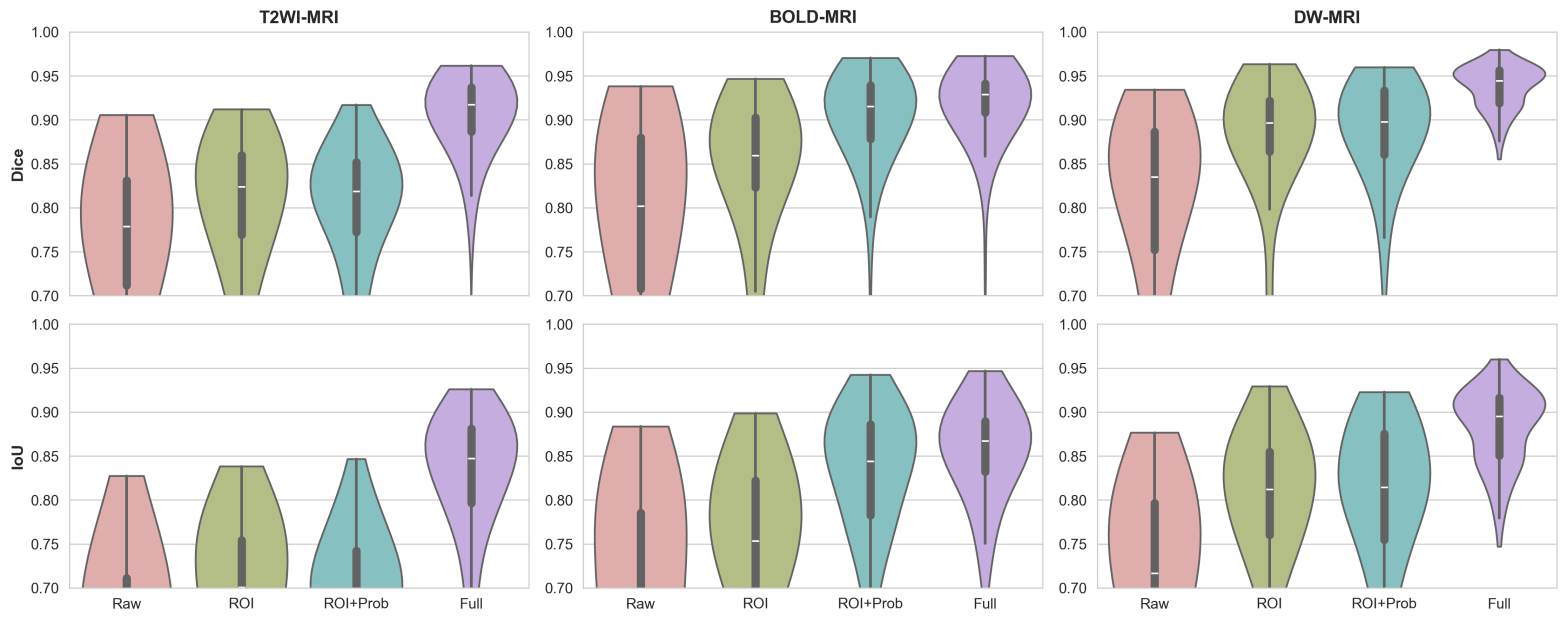
Dice (**top row**) and IoU (**bottom row**) score distributions across input configurations for the three MRI modalities under LOOCV, demonstrating progressive improvements in both mean performance and prediction consistency as each prior component is incorporated.

Four input configurations were evaluated to measure the contribution of each proposed component. In the first configuration, *Raw*, a grayscale original volume is directly fed into the network without any prior guidance and serves as the baseline. The second configuration, Region of interest *(ROI)*, applies kidney-centered spatial standardization to reduce background variability before passing the volume to the network. The third configuration, ROI and probability *ROI + Prob*, further augments the input with voxelwise probabilistic appearance maps derived in Section 3.2, forming a three-channel condition volume comprising the ROI as $c_{1}$ and the kidney probability map $P_{\mathrm{app}}^{K}{\left(I\right)}_{c}$ as $c_{2}$ and $c_{3}$. The fourth configuration, *Full Model*, replaces the last channel $P_{\mathrm{app}}^{K}{\left(I\right)}_{c}$ with the anatomical shape prior S of Section 3.3, forming the complete three-channel condition volume $c=[c_{1},c_{2},c_{3}]$. The qualitative evolution of the generated mask is illustrated in Figure 6, while the quantitative results for all configurations are summarized in Table 2 and visualized in Figure 7 to highlight the impact of each component on the final segmentation performance.

Table 2 shows that with each addition, there is a consistent and progressive improvement in the three modalities. At the same time, the raw grayscale baseline scores were consistently low mean Dice and high standard deviations regardless of the modality; this suggests the inability of the network to draw clearer kidney boundaries from intensity evidence, in particular, low contrast and inter-subject variability. ROI cropping improves Dice by 7.6–9.1% across modalities and does this by decreasing variation in the background content and by concentrating the network on the renal region. Then the addition of the probabilistic appearance maps gives a further substantial improvement in the mask quality shown in Figure 6, mostly evident in BOLD-MRI, where the Dice score increases from 83.17% to 90.71%, consistent with the multi-echo intensity heterogeneity inherent in this sequence that makes raw intensity distributions most uninformative for boundary localization. The full model incorporates the anatomical shape prior with even more improvement across all modalities as well as a big decrease in the standard deviation of Dice scores, showing that the shape prior is effective not only for mean accuracy but also for prediction consistency across subjects. In the full model, Dice scores of 90.86%, 92.02%, and 94.00% are accomplished on T2WI, BOLD-MRI, and DW-MRI, all above 90%, respectively, in all modalities. Figure 7 shows the Dice and IoU distribution for each configuration to underline the gradual enhancement and minimized inter-subject variability of Dice and IoU scores, which the addition of one component increased in all conditions, further confirms progressive improvement.

### 4.3. Comparison with State-of-the-Art Methods

Table 3 compares the full model setup of the proposed framework, defined as three-channel condition volume $c=[c_{1},c_{2},c_{3}]$ including the ROI volume, the voxelwise probabilistic appearance map, and the anatomical shape prior, relative to three competitive segmentation methods assessed using the same LOOCV protocol: UNEt TRansformers (UNETR) [34], a transformer-based architecture that represents long-range spatial dependencies; Segmentation-based Tissue Generative Adversarial Network (SegTGAN) [8], a GAN-based mechanism with densely interlinked generator structures and Segmentation Residual Network (SegResNet) [35], a purely convolutional residual architecture. Here, all models are retrained on the renal MRI data without using pre-trained weights. Hyperparameters were designed to account for the spatial resolution and volumetric dimensions of the kidney MRI scans.

Table 3 shows the quantitative comparison between the proposed approach and the state-of-the-art segmentation methods under the LOOCV protocol. On T2WI, the proposed model achieves the highest Dice score (90.86%) and demonstrates statistically significant improvements over all competing methods (p<0.05). The recorded results indicate that integrating the probabilistic appearance and anatomical priors provides a measurable advantage in low-contrast conditions, where boundary delineation is inherently challenging and intensity information alone is insufficient. On BOLD-MRI, the proposed framework also achieves the highest Dice score (92.03%) and shows statistically significant improvements over SegTGAN and SegResNet (p<0.05). While the margin improvement over UNETR is small, the overlapping confidence intervals suggest comparable performance, indicating that all high-performing models behave similarly under moderate contrast conditions. However, the proposed model shows consistent performance across subjects, reflecting its robustness to signal decay and susceptibility artifacts that are characteristic of multi-echo acquisitions. In contrast, the DW-MRI modality, where kidney-to-background contrast is naturally higher, SegResNet achieves a marginally higher Dice score (94.21%) compared with the proposed method (94.00%). This difference, while statistically significant, corresponds to a small effect size (r=0.27), suggesting limited impact under high-contrast conditions. This shows that the benefit of the proposed prior-guided framework becomes less pronounced when segmentation boundaries are already well-defined by image intensity. Table 3 overall suggests that the proposed approach provides the greatest benefit in challenging imaging conditions, particularly in low-contrast and heterogeneous MRI sequences such as T2WI and BOLD-MRI. This modality-dependent behavior emphasizes the role of explicit prior integration in improving segmentation reliability when image-based evidence is ambiguous.

**Figure 8:**
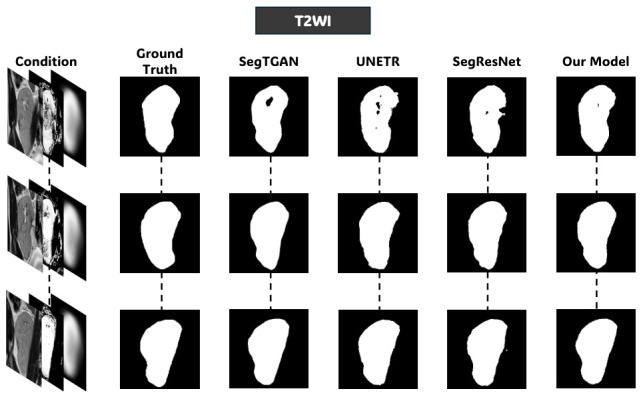
Qualitative comparison of kidney segmentation on T2WI. The input condition from the fourth configuration, ground-truth, and predicted masks by different models.

**Figure 9:**
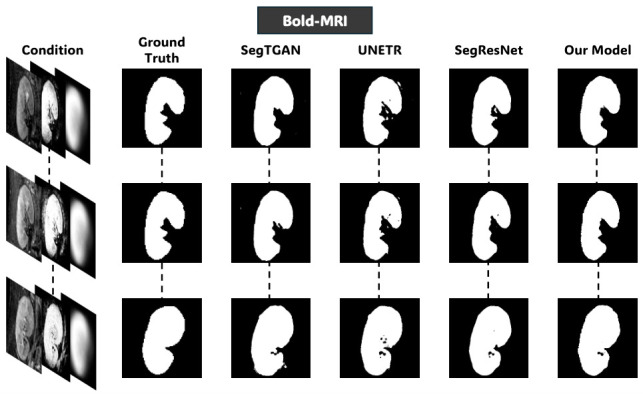
Qualitative comparison of kidney segmentation on Bold-MRI. The input condition from the fourth configuration, ground-truth, and predicted masks by different models.

**Figure 10:**
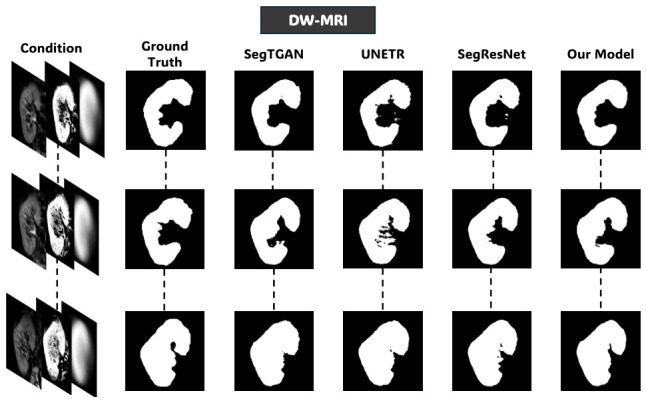
Qualitative comparison of kidney segmentation on DW-MRI. The input condition from the fourth configuration, ground-truth, and predicted masks by different models.

**Figure 11:**
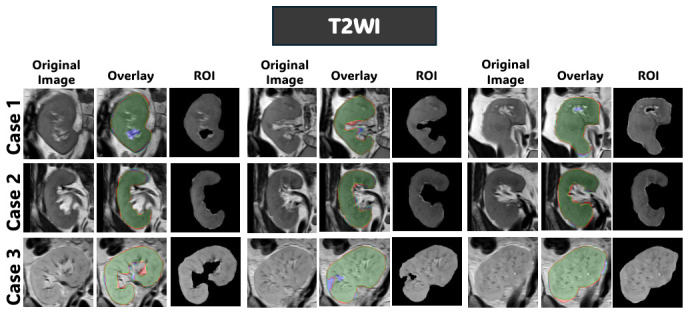
Qualitative segmentation results of the proposed model from configuration four on the T2WI dataset. For each case, the original image, segmentation overlay, and extracted kidney ROI are shown to illustrate the predicted volumetric masks. In the segmentation overlay, green indicates true positives (TP), red indicates false positives (FP), and blue indicates false negatives (FN).

**Figure 12:**
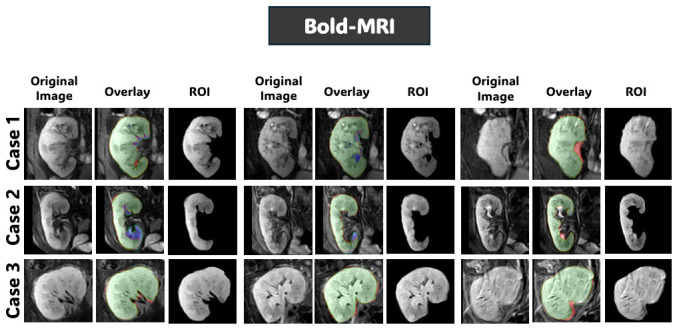
Qualitative segmentation results of the proposed model from configuration four on the Bold-MRI dataset. For each case, the original image, segmentation overlay, and extracted kidney ROI are shown to illustrate the predicted volumetric masks. In the segmentation overlay, green indicates true positives (TP), red indicates false positives (FP), and blue indicates false negatives (FN).

**Figure 13:**
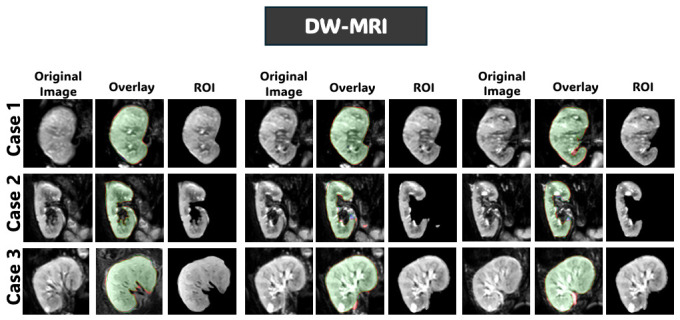
Qualitative segmentation results of the proposed model from configuration four on the DW-MRI dataset. For each case, the original image, segmentation overlay, and extracted kidney ROI are shown to illustrate the predicted volumetric masks. In the segmentation overlay, green indicates true positives (TP), red indicates false positives (FP), and blue indicates false negatives (FN).

Figure 8, Figure 9 and Figure 10 further support these observations. Figure 8 shows that on T2WI, the proposed approach achieves improved boundary delineation and more anatomically consistent kidney shapes in regions affected by low contrast. Figure 9 illustrates that on BOLD-MRI, the model maintains coherent structural predictions despite the artifacts and signal variations. Figure 10 shows that on DW-MRI, all methods produce accurate segmentation, with only minor boundary differences observed between approaches. Figure 11, Figure 12 and Figure 13 also illustrate that the proposed approach produces stable and anatomically consistent segmentation across all modalities. While small discrepancies remain in challenging regions for T2WI and BOLD-MRI, the overall segmentation quality remains high for biomarker extraction. In DW-MRI, the consistently strong performance across all methods reflects the reduced difficulty of the segmentation task under high-contrast conditions.

## 5. Discussion

The proposed framework achieves Dice scores between 90.86 and 94.01% across the three MRI modalities, a level of segmentation accuracy that is consistent with prior studies demonstrating reliable extraction of quantitative functional biomarkers from automatically segmented kidney masks [36,37]. The findings of this study suggest that prior guidance is valuable when the image evidence alone is inadequate for the delineation of anatomical boundaries. The enhanced performance of the proposed framework on T2WI and BOLD-MRI suggests an important limitation for purely data-driven segmentation methods in low-contrast MRI. In these modalities, the distribution of intensity between kidney tissue and its immediate tissues has too much overlap for networks that depend solely on image features to localize their boundaries accurately. The proposed framework allows for the inclusion of complementary structural information independent of raw image intensity, by conditioning the adversarial network on spatially localized appearance likelihoods aligned with a data-driven anatomical reference. This extra level of guidance helps the network tackle boundary ambiguities that cannot be convincingly derived from image evidence alone. Competing architectures on T2WI and BOLD-MRI can help further confirm this view, but demonstrate far inferior performance since these algorithms rely heavily on learned image features and tend to be affected in the same intensity ambiguities as these sequences.

SegResNet shows relatively slight advantages over the proposed framework for some DW-MRI boundary metrics. This observation may stem from differences in architectural regularization, rather than an inherent weakness of the prior-guided strategy, since both approaches work with the same data and evaluation conditions. In DW-MRI, provides the most reliable segmentation results among the investigated modalities. These observations stem from the enhanced kidney-to-background contrast due to high *b*-value diffusion weighting, which reduces the boundary ambiguity, enabling segmentation methods to localize the kidney more reliably. As a result, although purely image-driven architectures perform more competitively in this modality, the structural framework offered by the proposed priors continues to enhance prediction stability and anatomical plausibility. Conversely, T2WI and BOLD-MRI exhibit lower boundary contrast and greater signal variability, making kidney delineation more challenging. Consequently, the incorporation of structural constraints improves the stability of the segmentation.

An additional advantage of incorporating the anatomical shape prior is the reduction in inter-subject variability in the predicted masks. This reduction in variance occurs together with an improvement in the mean segmentation accuracy. This observation indicates that the prior does not simply constrain the predictions toward only a uniform kidney shape. Instead, it acts as a structural reference that discourages anatomically implausible predictions while still allowing natural morphological variation across subjects. By assigning lower prior support to regions outside the typical spatial footprint of the kidney, the framework suppresses inaccurate mask components and improves prediction stability in challenging cases.

From a clinical perspective, post-transplant graft monitoring currently follows a multimodality imaging approach in which duplex ultrasound serves as the first-line surveillance modality due to its accessibility, real-time capability, and low cost [38]. However, renal Doppler ultrasound is highly operator-dependent, and its interpretation can be challenging for practitioners with limited experience [39]. Furthermore, ultrasound cannot differentiate acute rejection from other causes of graft dysfunction, such as acute tubular necrosis or calcineurin inhibitor toxicity, necessitating invasive biopsy for definitive diagnosis [40]. Functional MRI techniques such as BOLD-MRI and DW-MRI address this gap by providing biomarkers that are reproducible, operator-independent, and sensitive to early functional changes preceding morphological deterioration [16,17,18]. T2WI, BOLD-MRI, and DW-MRI provide complementary physiological information related to morphology, tissue oxygenation, and microstructural diffusion, respectively, which form the basis for imaging biomarkers used to assess patients suspected of renal allograft rejection. Accurate and automated delineation of the transplant kidney is a prerequisite for consistent extraction of these biomarkers across modalities. The proposed framework directly addresses this clinical need by enabling robust segmentation under the heterogeneous and low-contrast conditions that characterize functional renal MRI, contributing toward reproducible noninvasive graft assessment that complements rather than replaces existing ultrasound surveillance protocols. In clinical practice, Doppler ultrasound-derived resistive index (RI) is commonly used as an indirect marker of graft perfusion, reflecting early post-transplant swelling and later fibrotic changes. However, RI is influenced by systemic hemodynamic factors and lacks spatial specificity [38,39]. In contrast, MRI-derived biomarkers obtained from accurately segmented kidneys provide spatially resolved and tissue-specific measurements, enabling more precise assessment of regional oxygenation and diffusion. This allows earlier detection of pathological changes and supports improved characterization of graft dysfunction beyond global indices such as RI.

Despite the promising results, this study has limitations to acknowledge. The data consists of 100 subjects, which were collected at a single institution with a single MRI scanner, which may limit data generalization to other scanners, field strengths, or acquisition protocols. Furthermore, 3D volumetric software has high computational requirements for training and inference, and such computational challenges can be a hurdle for realization at clinical facilities with low hardware resources. Lastly, the study population is specifically patients with kidney transplant, and there are no previous data on kidney pathologies such as native kidney disease, renal tumor, or polycystic kidney disease, as kidney morphology and imaging features can be greatly heterogeneous, thus still unknown to the effectiveness of the framework. Although LOOCV was employed to maximize training data per fold given the cohort size, it may yield optimistic variance estimates relative to repeated k-fold strategies; however, the statistical significance of reported improvements was validated through FDR-corrected Wilcoxon signed-rank tests on per-subject scores, confirming that observed gains are not attributable to variance inflation. Additionally, no formal a priori sample size estimation was conducted as the cohort size was determined by clinical data availability; nevertheless, 100 multisequence patients represent one of the largest reported cohorts for this specific task, and the consistency of results across all LOOCV folds with narrow confidence intervals supports the stability of the reported metrics.

## 6. Conclusions and Future Work

This study showed that guided probabilistic and anatomical priors integrated into a three-dimensional adversarial segmentation framework overcome the boundary ambiguity that characterizes functional renal MRI in kidney-transplant patients, especially in the low-contrast acquisition settings where data-only methods fall short. The proposed framework can achieve robust and reliable segmentation in three clinically distinct MRI modalities, demonstrating the utility of prior-conditioned adversarial learning for kidney-transplant evaluation. Among the investigated modalities, DW-MRI produced the most reliable segmentation results, which can be attributed to the high contrast between kidney and background tissues, facilitating boundary localization.

Future work will focus on external validation across multiple institutions and scanner platforms, generalization to other renal diseases beyond kidney transplantation, learning curve analysis for assessing performance scaling with larger training sets, and further exploring richer appearance priors that utilize the parametric information available in multi-echo and multi-*b*-value acquisitions to enhance boundary localization in challenging imaging settings.

## Figures and Tables

**Table 1:** MRI acquisition parameters for the three modalities (T2-weighted imaging (T2WI), blood-oxygen-level-dependent MRI (BOLD-MRI), diffusion-weighted MRI (DW-MRI)). TR/TE: repetition time/echo time.

Parameter	BOLD-MRI	T2WI	DW-MRI
In-plane resolution (mm)	1.25×1.25	0.63×0.63	0.72×0.72
Slice thickness (mm)	3	6	4
TR/TE (ms)	1330/16–80	670/80	4300/84
Special features	Multi-echo (5 TEs)	–	11 *b*-values (0–1000 s/mm^2^)

**Table 2:** Ablation study done on four input settings (Original raw data, Region of interest (ROI), ROI and probability (ROI + Prob), and full proposed model) under LOOCV (mean [95% CI]) on the three imaging modalities. Dice and Intersection Over Union (IoU) are reported in (%), while 95th Hausdorff Distance (HD95) and Average Symmetric Surface Distance (ASSD) are reported in (mm). ^†^ indicates that the Full Model significantly outperforms this configuration (Wilcoxon signed-rank test, FDR-corrected, p<0.05).

Dataset	Input Setting	Dice (%) ↑	IoU (%) ↑	HD95 (mm) ↓	ASSD (mm) ↓
T2WI	Raw	71.33 ^†^ [67.06, 75.59]	58.67 ^†^ [54.73, 62.62]	15.65 [10.63, 20.67]	4.00 [2.48, 5.52]
	ROI	78.93 ^†^ [76.40, 81.46]	66.63 ^†^ [63.85, 69.42]	7.56 [6.04, 9.08]	1.65 [1.21, 2.09]
	ROI + Prob	80.33 ^†^ [78.91, 81.74]	67.68 ^†^ [65.80, 69.55]	6.78 [5.28, 8.28]	1.28 [1.05, 1.51]
	Full Model	**90.86** [90.08, 91.64]	**83.47** [82.22, 84.72]	**2.07** [1.60, 2.54]	**0.41** [0.33, 0.49]
BOLD-MRI	Raw	74.51 ^†^ [70.48, 78.54]	62.65 ^†^ [58.43, 66.88]	11.78 [8.55, 15.01]	2.95 [1.78, 4.12]
	ROI	83.18 ^†^ [80.72, 85.64]	72.69 ^†^ [69.78, 75.60]	9.99 [7.28, 12.70]	1.71 [1.17, 2.25]
	ROI + Prob	90.72 ^†^ [89.82, 91.62]	83.31 ^†^ [81.86, 84.75]	2.53 [1.77, 3.29]	0.47 [0.36, 0.58]
	Full Model	**92.03** [91.31, 92.75]	**85.42** [84.26, 86.59]	**2.41** [1.43, 3.39]	**0.38** [0.26, 0.50]
DW-MRI	Raw	79.28 ^†^ [76.37, 82.19]	67.63 ^†^ [64.34, 70.92]	20.31 [13.48, 27.14]	3.86 [2.40, 5.32]
	ROI	87.82 ^†^ [85.98, 89.65]	79.19 ^†^ [76.94, 81.44]	6.37 [4.65, 8.09]	1.23 [0.97, 1.49]
	ROI + Prob	88.91 ^†^ [87.74, 90.08]	80.50 ^†^ [78.74, 82.26]	4.51 [3.32, 5.70]	0.83 [0.62, 1.04]
	Full Model	**94.01** [93.48, 94.49]	**88.76** [87.89, 89.63]	**1.60** [1.47, 1.73]	**0.31** [0.28, 0.34]

*Note:* Bold indicates the selected best-performing model at each metric. Higher Dice and IoU (↑) indicate better performance, while lower HD95 and ASSD (↓) indicate better boundary results.

**Table 3:** Comparison with state-of-the-art methods: UNEt TRansformers, Segmentation-based Tissue Generative Adversarial Network, Segmentation Residual Network under LOOCV (mean [95% CI]). Dice and Intersection Over Union (IoU) are reported in (%), while 95th Hausdorff Distance (HD95) and Average Symmetric Surface Distance (ASSD). ^†^ indicates that the proposed method significantly outperforms the competitor (Wilcoxon signed-rank test, FDR-corrected, p<0.05).

Dataset	Method	Dice (%) ↑	IoU (%) ↑	HD95 (mm) ↓	ASSD (mm) ↓
T2WI	UNETR [34]	87.46 ^†^ [86.53, 88.39]	78.01 ^†^ [76.61, 79.42]	2.99 [2.57, 3.42]	0.58 [0.52, 0.64]
	SegTGAN [8]	88.26 ^†^ [86.46, 90.06]	79.81 ^†^ [77.72, 81.89]	3.14 [2.26, 4.01]	0.63 [0.44, 0.82]
	SegResNet [35]	89.06 ^†^ [88.33, 89.79]	80.47 ^†^ [79.32, 81.61]	**1.47** [1.29, 1.65]	**0.26** [0.23, 0.30]
	Proposed	**90.86** [90.08, 91.64]	**83.47** [82.22, 84.72]	2.08 [1.60, 2.55]	0.42 [0.34, 0.50]
BOLD-MRI	UNETR [34]	91.92 [91.13, 92.70]	85.27 [84.01, 86.53]	2.45 [1.48, 3.41]	0.38 [0.27, 0.49]
	SegTGAN [8]	91.30 ^†^ [90.51, 92.09]	84.22 ^†^ [82.93, 85.51]	2.51 [1.73, 3.29]	0.46 [0.35, 0.56]
	SegResNet [35]	91.17 ^†^ [90.27, 92.07]	83.61 ^†^ [82.26, 84.95]	**1.86** [1.50, 2.22]	**0.36** [0.31, 0.41]
	Proposed	**92.03** [91.31, 92.75]	**85.42** [84.26, 86.59]	2.42 [1.42, 3.41]	0.39 [0.27, 0.51]
DW-MRI	UNETR [34]	93.95 [93.49, 94.40]	88.67 [87.88, 89.45]	1.57 [1.46, 1.69]	0.31 [0.28, 0.33]
	SegTGAN [8]	93.75 ^†^ [93.07, 94.09]	88.04 ^†^ [87.15, 88.92]	1.66 [1.51, 1.81]	0.34 [0.30, 0.37]
	SegResNet [35]	**94.21** [93.76, 94.66]	**89.14** [88.31, 89.94]	**0.54** [0.43, 0.64]	**0.11** [0.08, 0.15]
	Proposed	94.01 [93.51, 94.51]	88.80 [87.93, 89.66]	1.60 [1.44, 1.71]	0.31 [0.28, 0.35]

*Note:* Bold indicates the selected best-performing model at each metric. Higher Dice and IoU (↑) indicate better performance, while lower HD95 and ASSD (↓) indicate better boundary results.

## Data Availability

The datasets used and/or analysed during the current study are not publicly available because access is limited to appropriately trained individuals. Data may be made available from the corresponding author upon reasonable request and subject to applicable institutional, ethical, and data-use requirements.
